# Human Respiratory Infections in Nigeria: Influenza and the Emergence of SARS-CoV-2 Pandemic

**DOI:** 10.3390/vaccines10091551

**Published:** 2022-09-17

**Authors:** Dennis Kabantiyok, Nathaniel Ninyio, Ismaila Shittu, Clement Meseko, Theophilus I. Emeto, Oyelola A. Adegboye

**Affiliations:** 1 Laboratory Diagnostic Services Division, National Veterinary Research Institute, PMB 01, Vom 930001, Nigeria; 2 School of Medical Sciences, Örebro University, 70182 Örebro, Sweden; 3 Department of Avian Influenza and Transboundary Animal Diseases, National Veterinary Research Institute, PMB 01, Vom 930010, Nigeria; 4 Public Health & Tropical Medicine, College of Public Health, Medical and Veterinary Sciences, Department, James Cook University, Townsville, QLD 4811, Australia; 5 World Health Organization Collaborating Center for Vector-Borne, Neglected Tropical Diseases Department, College of Public Health, Medical and Veterinary Sciences, James Cook University, Townsville, QLD 4811, Australia; 6 Australian Institute of Tropical Health and Medicine, James Cook University, Townsville, QLD 4811, Australia

**Keywords:** SARS-CoV-2, influenza, Nigeria, pandemic

## Abstract

The increasing outbreak of zoonotic diseases presents challenging times for nations and calls for a renewed effort to disrupt the chain of events that precede it. Nigeria’s response to the 2006 bird flu provided a platform for outbreak response, yet it was not its first experience with Influenza. This study describes the impact of SARS-CoV-2 on Influenza surveillance and, conversely, while the 1918 Influenza pandemic remains the most devastating (500,000 deaths in 18 million population) in Nigeria, the emergence of SARS CoV-2 presented renewed opportunities for the development of vaccines with novel technology, co-infection studies outcome, and challenges globally. Although the public health Intervention and strategies left some positive outcomes for other viruses, Nigeria and Africa’s preparation against the next pandemic may involve prioritizing a combination of technology, socioeconomic growth, and active surveillance in the spirit of One Health.

## 1. Introduction

Zoonotic respiratory viruses are responsible for more disease outbreaks at the human-animal interface than any other source [1]. The National Institute of Health (NIH) reported that over 18% of emerging and re-emerging zoonotic diseases are caused by respiratory viruses such as the Severe Acute Respiratory Syndrome Coronavirus 2 (SARS-CoV-2), Middle East Respiratory Syndrome Coronavirus (MERS-CoV) and influenza [2]. As a result of global warming, rapidly changing ecology, increased global travel, population growth, urbanization and increasing human disruption to the wild, the world is likely to experience increasing emergence and re-emergence of zoonotic pathogens [3,4,5]. These factors interplay in a complex manner that challenges public health response in mitigating infectious disease spread. There are speculations that the novel SARS-CoV-2 emerged due to the growing desire for bushmeat in Asia [6,7,8,9]. The extensiveness of disease outbreaks and their convoluted spread in the environment, animals, and humans necessitated the World Health Organization’s (WHO) Tripartite One Health approach to address future outbreaks and improve surveillance at the human-animal interface [10]. At the centre of the tripartite approach is the Food and Agricultural Organization (FAO), World Organization for Animal Health (WOAH) and WHO partnership to address challenges in humans, animals, and environmental health through a multisectoral and multidisciplinary approach [11], bearing in mind that health cannot be achieved by working in silos.

In this study, we provide a brief overview of outbreaks of influenza and SARS-CoV-2 outbreaks in Nigeria by conducting a systematic review of articles on the subject. The intent is to unravel the effect of the SARS-CoV-2 outbreak on Influenza status. We also highlighted how dealing with the influenza outbreak modified the country’s response to SARS-CoV-2. We show how the Nigeria influenza virus experience shifted from humans to animals, particularly avian influenza, and the lessons learned from the perspective of SARS-CoV-2 in Nigeria.

## 2. Materials and Methods

Ninety-five articles were extracted between January and June 2022. Literature search focused on the following keywords; influenza, avian influenza, pandemic preparedness, COVID-19, COVID-19 surveillance, and COVID-19 in Africa by using search engines such as Google Scholar, Science Direct, Nature, Scopus, PubMed, and Web of Science. Inclusion criteria include research articles, review articles, technical reports, newspaper reports and guidelines written in English.

## 3. Historical Overview of Influenza in Nigeria

Early studies on influenza in Nigeria date back to the seroprevalence of influenza A during the 1918 Spanish flu [9,12]. While the virus’s origin remains debatable, its entry into Nigeria was likely due to interaction with the colonialist and importation through the Great War (World War 1) veterans [12]. The 1918 pandemic remained Nigeria’s first documented and most deadly pandemic affecting 50% to 80% of the population and killing 500,000 in a population of 18 million Nigerians [13]. The 1918 flu pandemic crippled Nigeria’s economy [13]. Among other factors, the fear-motivated movement of persons from major epicentres to the countryside and the trans-regional trade networks facilitated by railways across the country were instrumental in furthering the spread of the virus [14]. Aside from the 1918 pandemic, Nigeria has continued to experience seasonal flu outbreaks of lesser magnitudes [15]. For example, the 1974 epidemic was confirmed via egg isolation and a hemagglutination–inhibition test in which thirteen influenza strains were identified with high seroconversion (80–95%) of A/Nigeria/1/74 among persons tested [15]. 

Although early data on influenza in Nigeria showed devastating effects on humans [13,15], the virus has considerably shifted focus to animals, particularly birds [16,17,18,19], over the last three decades. This is backed by a growing agribusiness [20] and the role of migratory waterfowl as the natural reservoir and global distributors of influenza [21,22]. The shift in influenza epidemiology to animals reflects the need for a One Health approach by employing active surveillance at the human–animal interface and increasing awareness to search and identify early warning signals for a potential pandemic. Advancements in vaccinology, host-pathogen interaction, laboratory investigations and effective public health strategies may have expanded the need for human-animal inclusiveness at the global level. Nigeria’s interest in animal influenza research was triggered by its negative economic consequence on poultry [22,23,24]. This is because the poultry industry is a fast-growing sector and an effective tool for poverty alleviation in Nigeria [25]. Farmers’ losses attributed to the 2006 bird flu cost the nation an estimated revenue loss of more than USD 5.4 million [26], while the compensation paid by the government to the affected farmers is estimated at USD 11.5 million [27]. The Nigerian government’s response to the avian influenza outbreak led to active surveillance and inter-ministerial collaboration in the One Health approach [28]. Nigeria’s position as a hotspot for influenza in Africa necessitated a national action plan for influenza pandemic preparedness [29,30]. Although the document was yet to be effected in an influenza pandemic situation, it was evoked as part of the pre-outbreak preparedness following the emergence of SARS-CoV-2 [31].

Although Nigeria set up its WHO African Region’s Integrated Disease Surveillance and Response Strategy (IDSRS) in 2001 [32], it was not robust enough on human influenza data. However, in 2008, a renewed interest in human influenza was necessitated by the likelihood of zoonotic spillover from avian bird flu in 2006 [33]. In collaboration with four other sentinel laboratories spread across the country, the WHO national reference lab conducted a large survey on human influenza in Nigeria. The surveillance, which lasted from April 2009 to August 2010, found a prevalence of 7.7% (217/2803) among persons presenting influenza-like illnesses (ILI) and severe acute respiratory illnesses (SARI) [33]. Surveillance activities in humans have become imperative due to the rising frequency of bird flu outbreaks in Nigeria and concerns that the virus might evolve antigenically to adapt to human hosts [34]. 

In 2015, there was a re-emergence of Highly Pathogenic Avian Influenza (HPAI) [35], and in 2021 the introduction of a Eurasian clade (2.3.4.4b) of influenza into Nigeria and Senegal [36,37]. These outbreaks occurred in January, and the role of migratory birds has been highlighted as a possible source. Later in May, Lesotho reported the same clade of HPAI was causing epizootic outbreaks. The Lesotho clade showed 98.93–99.93% nucleotide identity with the Nigerian and Senegalese clade [38]. 

### 3.1. The Emergence of SARS-CoV-2 in Nigeria

Before the 2019 pandemic, studies have shown the existence of coronaviruses circulating in the wild, especially in Bats [39,40,41]. While other coronaviruses have been shown to have a global presence [42], it was the SARS-CoV-2 pandemic that brought it to the limelight in Nigeria. The novel SARS-CoV-2 was first detected in an Italian traveller in Nigeria on the 27 February 2020 [43]. Prior to that, Quan et al., 2010 [41] reported the discovery in 2010 of a novel coronavirus from Commerson’s leaf-nosed bat (*Hipposideros commersoni*) in Zaria (ZBCoV) 2010 was the first of its kind in Nigerian wildlife, and it positioned Nigeria on the map of coronaviruses endemic countries. Similarly, in another study in 2016, Stefania et al. [44] detected and identified Betacoronavirus from fruit bats in Ibadan, Nigeria, 2016.

Although countries have now approved guidelines for the treatment of SARS-CoV-2 [45], conflicting information, such as inconsistent scientific findings on treatment and differing recommendations among professionals, were common in the early stages of the pandemic. This was expected because knowledge of the virus was incremental [46,47], although these missteps negatively impacted public perception of the virus. For example, there was no unified guideline on the use of nose masks [48], and this broods more controversy. Other drawbacks unique to the Nigeria setting include; ineffective distribution of relief materials, inadequate healthcare facilities, adoption of foreign containment measures that do not align with domestic economic realities, misleading information from public figures and erroneous belief that the pandemic affects the wealthy who have a high propensity to travel oversea [49,50,51].

The Nigerian government’s public health measures/guidelines, such as restrictions on foreign travel and lockdown of major cities, have kept the local transmission in check, as evidenced by the slow epidemic trajectory in the first month [52]. Aside from these measures, before the arrival of the index case, the government had upgraded testing capacity and commissioned a laboratory network, provided isolation centres for case management, set up the Ministerial Expert Advisory Committee on COVID-19 (MEACoC), case management, infection prevention and control, and risk communication as pandemic preparedness [53]. As a novel disease, nations had to rely on new information that guided policies on public health. Some of these adopted interventions, such as ‘stay at home’ and quarantine, were disproportionately borne by those who were already disadvantaged [54]. Nonetheless, the early approach to containment has been effective, albeit it came at the expense of people’s livelihood, education and mental health [55]. 

### 3.2. The Impact of COVID-19 on Respiratory Viruses in Nigeria

Due to the similarity in symptoms displayed by other self-limiting respiratory infections such as the common cold, the perception of COVID-19 in Nigeria has been rather simplistic and counterproductive to government restrictions aimed at mitigating its spread [56]. Globally, respiratory outbreaks have been shown to influence seasonal diseases in unique ways because interventions and public health measures in curtailing the outbreaks, such as respiratory hygiene, handwashing, and social distancing, have a ripple effect on other diseases. For example, Yuan et al., 2021 [57] showed a significant reduction in endemic respiratory viruses such as influenza, human coronaviruses (CoV-OC43, CoV-229E), parainfluenza (PIV-2, PIV-4,) and respiratory syncytial virus (RSV). Moreover, several other studies have reported a significant drop in endemic seasonal respiratory viruses in Australia [58], New Zealand [59], China [60], Canada [61], Chile and South Africa [62], thereby further bolstering that COVID-19 interventions produced overlapping positive outcomes for other respiratory viruses [63]. In another study, Swets et al., (2022) reported that co-infection of COVID with influenza ranked high among other viruses in England. However, co-infection of COVID-19 with influenza and adenoviruses was significantly associated with death [64]. Despite the evidence of co-infection of COVID-19 with other respiratory viruses, such as influenza, and RSV [64,65,66], surveillance of influenza in humans in Nigeria is limited.

### 3.3. Rising Threats of Novel Zoonotic Spill-Over

The drivers of zoonotic outbreaks are intertwined in a web of complex events, making it impossible to understand one facet independent of the other. Before the 2019 pandemic, there has been growing evidence of the close relationship between human health and a healthy ecosystem [67]. The rising zoonotic disease outbreaks reflect a recent tapering interface between humans and animals. Several factors such as man’s increasing encroachment into the wild, illegal wildlife trade, rising global population and food insecurity, and disruption of the ecosystem through urbanization may have significantly contributed to spillover events [68,69,70,71]. On the other hand, the fast pace of technological advancement facilitates the rapid spread of outbreaks since the time it takes to travel between continents is shorter than the incubation period of most pathogens. This means nations must grapple with the consequences of emerging and re-emerging pathogens [72]. 

The last two decades have witnessed several zoonotic outbreaks. Notably, SARS-CoV in 2003, which originated from bats and civets, Highly Pathogenic Avian Influenza (HPAI) H5N1 in 2002 and 2006, and Middle East Respiratory Corona Virus (MERS-CoV) in 2014 from dromedary camels, Ebola outbreak from bats in Western Africa in 2014, re-emergence of Monkeypox virus in 2017 and 2022, and the recent SARS-CoV-2 in 2019 [73,74]. An important risk factor synonymous with these outbreaks is the unregulated wildlife trade. However, the wildlife trade has been linked to disease outbreaks in humans and livestock, leaving substantial economic losses in international trade [75,76]. Food insecurity, poverty, cultural practices, and corruption remain major drivers of the wildlife trade. This emphasizes the socioeconomic dimension of zoonotic disease. Hence, there is a higher risk of zoonotic spillover from areas where the wildlife trade is rife.

### 3.4. COVID-19 in Africa: Pandemic Preparedness in Diagnostics, Case Management and Vaccination

A critical step in pandemic preparedness is effective disease surveillance. COVID-19 reinforced that disease surveillance measures in most African nations are inadequate due to diagnostic insufficiency, geographical/cultural barriers, and poor data management [77]. For instance, epidemics such as Ebola have been actively spreading for over a month before being detected [78]. Although travel and movement were restricted in many countries at the onset of the COVID-19 pandemic, there is little evidence to suggest that these restrictions were imposed early enough or strictly enough across Africa. For example, the initial airport surveillance in Nigeria, Ivory Coast and Egypt focused on individuals with recent travel history to China [79]. However, recent genomic surveillance demonstrated how COVID-19 was predominantly initiated in Africa via trade-related interactions and importations from Europe. The study reported about 757 transcontinental introductions of the SARS-CoV-2 into Africa between 2020 and 2021 [79]. Taken together, this suggests that disease surveillance towards pandemic preparedness needs to be expansive and continuous. The major drawback to more effective disease surveillance in many African nations is inadequate funding for these countries’ health and research sectors. Kapiriri et al. [80], studied the pandemic preparedness plan of 18 African nations and highlighted that pandemic preparedness was not generally considered a priority. The countries have various needs in their budgets that must compete for the limited funds.

In addition to disease surveillance, public health education is crucial to pandemic preparedness. However, the learning crisis, absence of compulsory education, and low school completion rates in most African countries pose a significant challenge to the use of education in pandemic preparedness [81]. The COVID-19 pandemic highlighted the impact of education, or the lack thereof, on upholding or endangering public health. For example, students of health professions in sub-Saharan Africa demonstrated adequate knowledge of COVID-19 transmission and prevention because public health is included in their curricula [82]. This demographic, however, only represents a small population of the entire African populace. Therefore, it is necessary to educate non-health professions students on the rudiments of public health as a means of pandemic preparedness. Additionally, intensive public health awareness which is aimed at educating people with no formal education is a critical approach to pandemic preparedness in Africa. Another study on selected countries in south, east and west Africa showed that even where these were practised initially, a decline in adherence to handwashing as the COVID-19 pandemic progressed was prevalent [83]. A similar occurrence was also reported in Hong Kong, where a reduction in public adherence to mask-wearing, social distancing, and handwashing was recorded. In all of the aforementioned cases, the decline in adherence was lower in individuals with higher education.

Another essential aspect of pandemic preparedness is how stakeholders in the public health sector can be more effective. These stakeholders include virologists, bacteriologists, immunologists, vaccinologists, nurses, physicians, epidemiologists, pharmacists, etc. When there is a call to action, it is expected that highly functional research and development facilities be available to ease effective response to pandemics [84,85]. Compared to the rest of the world, Africa has one of the weakest healthcare sectors, whose response has been slow and disproportionate to the severity of the COVID-19 pandemic [86]. Furthermore, when cutting-edge health and medical research is encouraged and funded in a nation, important insight into common diseases will be gained, consequently enhancing pathogen predictability and the development of effective vaccines and agents [87]. Viral predictability has been an important tool in disease control and pandemic preparedness to control influenza outbreaks [88,89] effectively. 

The role of vaccines in mitigating the spread of COVID-19 cannot be overemphasized. While the development of the COVID-19 vaccine was hinged on an entirely new technology based on mRNA, a pre-existing infrastructure for vaccine development by big Pharmaceutical companies in the West and the need to curb the spread facilitated the rapid vaccine design [90]. In Africa, asides from Egypt and South Africa, no other country is involved in manufacturing the vaccine, thus contributing to poor vaccine nationalism [91]. Nigeria’s weak health security means stockpiling vaccines was impossible, so donations from foreign allies and philanthropists form a major source of vaccine acquisition. Eventually, it became clear that having the vaccine was not enough as nations had to deal with other drawbacks which affected vaccine acceptance. For a pandemic buried in suspicion, the government will have to manage viral infodemic and several other conspiracies negating control strategies [92]. Although illiteracy has been suggested as a key driver for vaccine hesitancy [93], developed countries, such as France and the US, with higher literacy levels, experienced vaccine hesitancy [94]. This directly correlates with how trusting a populace is of her government. For example, Pak et al. [95] showed that high levels of public trust and perception of truthfulness in government often result in a significant increase in the number of people willing to comply with government policies. 

## 4. Conclusions

In this review, we have provided a brief overview of influenza and SARS-CoV-2 in Nigeria by systematically appraising relevant articles to highlight the Nigerian response to COVID-19 and unravel the effect of SARS-CoV-2 on other respiratory viruses, especially influenza. The emergence of avian influenza in Nigeria had a huge economic impact on the poultry business patronized by small-scale farmers who constitute the vast majority of poultry businesses. We believe that the 2006 avian influenza outbreak provided Nigeria with some level of pandemic preparedness which was useful in the COVID-19 era, although the effect of such experience needs to be empirically measured, the paucity of data on research into the human influenza virus in Nigeria is a major drawback. As a result, while there have been reports of a drop in the seasonal cases of other respiratory viruses globally, there is no compelling evidence to support such proof in Nigeria. 

The COVID-19 pandemic exposed a weak healthcare system in many developing countries that are already battling other perennial health issues (HIV/AIDS, Tuberculosis, and Malaria). The response to disease outbreaks will continue to be challenging especially in low-resourced countries, hence, there is a need for both localised and global control measures in the event of future outbreaks to fit current socioeconomic and psychosocial realities combined with an efficient health system. There is also the need for surveillance on the animal–human interface because antigenic transformations in avian influenza and other viruses that favour transmission to humans remain a potential threat to global health security. 

## Data Availability

The data presented in this study are openly available in the reference section.
